# Supplementary material for: Effects of Socio-Familial Behavior on Sleep Quality Predictive Risk Factors in Individuals under Social Isolation
Source: Int J Environ Res Public Health. 2022 Mar 20;19(6):3702. doi: 10.3390/ijerph19063702 (PMC8950965; doi:10.3390/ijerph19063702)
Supplement: Supplementary file 1 [file ijerph-19-03702-s001.zip › ijerph-1628275-supplementary.pdf]

SLEEP and FAMILY perceptions during the outbreak COVID-19

Good day.

THIS QUESTIONNAIRE IS INTENDED FOR ONLY THOSE PEOPLE WHO ARE IN A SITUATION OF VOLUNTARY ISOLATION.

Please DO NOT fill out this questionnaire if you:

1.HAVE BEEN DIAGNOSED BY RESPIRATORY DISEASE DERIVED FROM COVID-19, AND YOU ARE UNDER MANDATORY ISOLATION.

2. HAD PREGNANCY LABOR AT LEAST 6 MONTHS AGO.

The Autonomous University of Baja California and CETYS University have launched an investigation into Perceptions of Behavior during an outbreak by COVID-19. Both Universities are in Mexico and do not present any political interest for the realization of this study.

Completing this survey will take 15 minutes.

Your responses are very important to better understand the problems that families are going through in their homes and the way they may increase, by the current situation of isolation derived from the expansion of COVID-19, as well as to anticipate prevention measures for the following weeks.

Please answer as sincerely as you can, the completion of this questionnaire is voluntary and anonymous, at no time will you be asked for identification information but an e-mail for possible follow-up.

Try to answer all questions except those that do not apply to your actual situation.

Finally, we want to tell you that if we successfully receive your answers, within 30 days you will receive a second version of this questionnaire as a follow-up to this study.

Thank you!

*Obligatorio

1. I accept to participate voluntarily and anonymous by filling this questionnaire when marking the "yes" box. *

Selecciona todos los que correspondan.

- [ ] Yes
- [ ] No

Personal information

In this section, please help us identify some basic aspects of your personal life.

2. 1. Due to COVID-19, do you currently ... *

Marca solo un óvalo.

- ( ) I go out MORE than before.
- ( ) I go out the same as before and go about my normal life.
- ( ) I've taken precautionary measures, but I'm still going out.
- ( ) I have stopped going out, but family or friends come and go from home.
- ( ) No one leaves the house, nor are visitors accepted, and only I or a family member leaves for food.
- ( ) No person enters or leaves, everything is requested at home.
- ( ) I am in total quarantine, nothing enters or leaves, I sustain myself with previously acquired resources.

3. 2. Thinking about the previous answer, how many days have you been in this state? *

____

4. 3. In which country do you LIVE? *

____

5. 3.1 In which country were you BORN? *

6. 4. In which city do you LIVE? *

7. 4.1 In which city did you BORN? *

8. 5. Your age in years is: *

9. 6. You are a: *

Marca solo un óvalo.

- Male
- Female
- Prefer not to say
- Otro: ____________

10. 7. What is your religion? *

11. 8. Civil status *

Marca solo un óvalo.

- Single
- Married
- Free union
- Divorced
- Widower

12. 9. Do you have health insurance? *

Marca solo un óvalo.

- Yes
- No

13. 10. If you have health insurance that is:

Marca solo un óvalo.

- Private
- Public
- Both

14. 11. Are you a student? *

Marca solo un óvalo.

- Yes
- No

15. 12. What is your last degree? *

Marca solo un óvalo.

- Elementary School
- Junior Highschool
- Highschool
- Undergrade
- Graduate
- Master degree
- Doctoral PhD

16. 13. Do you smoke? *

Marca solo un óvalo.

- Yes
- No

17. 14. If you answered "yes" to the previous question please indicate quantity and frequency.

____

18. 15. Do you drink any kind of alcoholic beverage? *

Marca solo un óvalo.

- Yes
- No

19. 16. If you answered "yes", how many times did you drink during the last month?

____

20. 16.1. Of those times you drink how many cans/pots or cups/glasses did you drink?

____

21. 17. Do you consider that the amount of your alcohol consumption has increased as a result of voluntary isolation? *

Marca solo un óvalo.

- Yes
- No

22. 18. Do you use any type of drug (besides tobacco and alcohol)? *

Marca solo un óvalo.

- Yes
- No

23. 19. If you answered "Yes" to the previous question, how many times did you consume them in the last month?

____

24. 19.1. Of those times that you consumed it, how many units did you consume?

____

25. 20. Do you drink energy drinks (coffee, carbonated drinks, etc.)? *

Marca solo un óvalo.

- Yes
- No

26. 21. What is your weight in pounds?

____

27. 22. What is your height in feet-inches?

____

28. 23. Please indicate the people with whom you live and your relationship, you can indicate more than one box. *

Selecciona todos los que correspondan.

- [ ] Alone
- [ ] Family (couple, partner and children, dad, mom and brothers)
- [ ] Couple
- [ ] Second relatives (brothers, brothers-in-law, in-laws, etc.)
- [ ] Others (friends, or people you know)

29. 24. If you have sons or daughters, how old are they?

____

30. 25. Do you work? *

Marca solo un óvalo.

- Yes
- No

31. 26. What do you consider to be your socioeconomic level? *

Marca solo un óvalo.

- Low
- Middle
- High

32. 27. Your income comes from ... *

Selecciona todos los que correspondan.

- I have a fixed salary
- I have my own business

Otro:

33. 28. Has the current situation of isolation caused you concerns about your financial income? *

Marca solo un óvalo.

- Yes
- No

34. 29. Has the current situation of isolation unbalanced the economy of your home? *

Marca solo un óvalo.

- Yes
- No

35. 30. Has the current situation of isolation caused you to have less food in your home? *

Marca solo un óvalo.

- Yes
- No

36. 31. What means of communication do you use most frequently to receive information about the situation of the pandemic? *

Selecciona todos los que correspondan.

- Television news and / or print media
- Medios oficiales de autoridades sanitarias (informe diario de secretaría de salud, páginas de internet oficiales, etc.)
- Official means of health authorities (daily report of the health department, official internet pages, etc.)
- Through other people (family, friends, acquaintances)
- I am not in contact with information about the situation

Otro: ______________________

37. 32. How much do you agree with the voluntary isolation measures? *

Marca solo un óvalo.

	1	2	3	4	5	
Absolutely disagree						Completely agree

38. 33. How often have you been going outside during the period of voluntary isolation? *

Marca solo un óvalo.

- No day during the week.
- 1 day a week.
- 2 to 3 days a week.
- Everyday during the week.
- Otro: ____________________

Self-perceived sleep behavior

Please answer according to your perception regarding your way of sleeping.

39. 1. In the past month, have you been diagnosed with some medical illness that relates with sleep disorders (mostly mental health issues such as depression or anxiety)? *

Marca solo un óvalo.

- Yes
- No

40. 2. If you answered “yes” to the previous question, please specify which one.

____

41. 3. Do you take any sleeping medication?

Marca solo un óvalo.

- Yes
- No

42. 4. Since the beginning of the outbreak, do you consider that your sleep habits have changed? *

Marca solo un óvalo.

- Yes
- No

43. 5. If you answered “yes” to the previous question, please state what you think the sleep problems could be associated with:

Selecciona todos los que correspondan.

- [ ] Labor inactivity
- [ ] Anxiety for your health situation or your family’s
- [ ] Economic uncertainty
- [ ] Social isolation
- [ ] All of the above

Otro: [ ] ______________________

44. 6. Please specify the average number of hours you regularly sleep each night. *

____

45. 7. Do you usually drink carbonated drinks (example: soda or mineral water)? *

Marca solo un óvalo.

- Yes
- No

46. 8. If you answered “yes” to the previous question, please specify the quantity and frequency.

Marca solo un óvalo.

- 1 to 3 units per week.
- 1 to 3 units per day.
- 4 to 6 units per day.
- More than 7 units per day.

47. 9. Since the beginning of the outbreak, do you think that the amount of energy or carbonated drinks has increased? *

Marca solo un óvalo.

- Yes
- No

48. 10. Since the beginning of the outbreak, do you think that the amount of food you eat during the evenings has increased? *

Marca solo un óvalo.

- Yes
- No

49. 11. Since the beginning of the outbreak, have you noticed that you go to bed to sleep, later than days before? *

Marca solo un óvalo.

- Yes
- No

50. 12. If you answered "Yes" to the previous question, please specify.

Marca solo un óvalo.

- 1 to 2 hours later.
- 2 to 4 hours later.
- 4 to 6 hours later.
- I have risen without being able to fall aslep.

51. 13. Specify the approximate time you go to bed, during the week, since the outbreak began. *

Ejemplo: 8:30 a.m.

52. 14. Specify the approximate time you wake up, during the week, since the outbreak began. *

Ejemplo: 8:30 a.m.

53. 15. Specify the approximate time you go to bed on weekends since the outbreak began. *

Ejemplo: 8:30 a.m.

54. 16. Specify the approximate time you wake up, on weekends, since the outbreak began. *

Ejemplo: 8:30 a.m.

55. 17. Since the beginning of the outbreak, has the average number of hours that you slept changed? *

Marca solo un óvalo.

- Yes
- No

56. 18. Since the beginning of the outbreak, have you started napping when you didn't before? *

Marca solo un óvalo.

- Yes
- No

57. 19. How many hours (including nap times) do you currently sleep on weekends per day? *

____

58. 20. Do you consider yourself a night working person (works or concentrates better at night), or daytime (works or concentrates better during the day)? *

Marca solo un óvalo.

- Night working
- Daytime working

59. 21. Since the beginning of the outbreak, have you perceived that you wake up at night to go to the restroom or for no reason? *

Marca solo un óvalo.

- Yes
- No

60. 22. If you answered “yes” to the previous question, is it easy for you to fall asleep again?

Marca solo un óvalo.

- Yes
- No

61. 23. Since the beginning of the outbreak, do you feel rested when you wake up? *

Marca solo un óvalo.

- Yes
- No
- I usually take naps from time to time.

62. 24. As a result of voluntary isolation, how do you consider your quality of sleep? *

Marca solo un óvalo.

- Very good
- Good
- Regular
- Bad
- Very bad

Personal self and family perceived behaviors

The following questions are related to how you feel at home in the current situation, compared to your normal life habits prior to the outbreak. Please indicate how much you agree or disagree with each of the statements presented by choosing from the following:

1. In disagree
2. Slightly disagree
3. Slightly agree
4. Strongly agree

Personally, facing this outbreak has caused...

Please complete according to your perception.

63. SF1.1. an increase in my angry reactions. *

Marca solo un óvalo.

	1	2	3	4	
In disagree	◯	◯	◯	◯	Strongly agree

64. SF1.2. the feeling of losing control of my actions. *

Marca solo un óvalo.

	1	2	3	4	
In disagree	◯	◯	◯	◯	Strongly agree

65. SF1.3. a change in my hopes for the future. *

Marca solo un óvalo.

	1	2	3	4	
In disagree	◯	◯	◯	◯	Strongly agree

66. SF1.4. a loss of motivation to do the things I must do on a daily basis. *

Marca solo un óvalo.

	1	2	3	4	
In disagree	⬭	⬭	⬭	⬭	Strongly agree

67. SF1.5. a decrease in my ability to pay attention and focus clearly. *

Marca solo un óvalo.

	1	2	3	4	
In disagree	⬭	⬭	⬭	⬭	Strongly agree

68. SF1.6. me to try to generate new solutions to my problems. *

Marca solo un óvalo.

	1	2	3	4	
In disagree	⬭	⬭	⬭	⬭	Strongly agree

At the family level, facing the outbreak...

Please complete according to your perception.
If you live alone, please go to the next sections and finish the questionnaire.

69. SF2.1. has increased conflicts within my family.

Marca solo un óvalo.

	1	2	3	4	
In disagree	⬭	⬭	⬭	⬭	Strongly agree

70. SF2.2. has increased conflicts with my partner.

Marca solo un óvalo.

	1	2	3	4	
In disagree	⬭	⬭	⬭	⬭	Strongly agree

71. SF2.3. has increased my family's collaboration regarding carrying out household chores.

Marca solo un óvalo.

	1	2	3	4	
In disagree	⬭	⬭	⬭	⬭	Strongly agree

Children's behaviors

Please complete according to your perception.
If you live alone, please go to the next sections and finish the questionnaire.

72. SF3.1. I find easy to identify when my child(ren) is(are) concerned or worried.

Marca solo un óvalo.

	1	2	3	4	
In disagree	⬭	⬭	⬭	⬭	Strongly agree

73. SF3.2. I usually notice about the situations which cause anxiety or stress to my child(ren).

Marca solo un óvalo.

	1	2	3	4	
In disagree	⬭	⬭	⬭	⬭	Strongly agree

74. SF3.3. I can resolve or lessen conflicts that arise with my child(ren) without making them more complicated.

Marca solo un óvalo.

	1	2	3	4	
In disagree	⬭	⬭	⬭	⬭	Strongly agree

75. SF3.4. Most of the time I can keep control of the situation with my child (ren) at home.

Marca solo un óvalo.

	1	2	3	4	
In disagree	⬭	⬭	⬭	⬭	Strongly agree

76. SF3.5. It has been easy for me to keep schedules organized for different activities at home.

Marca solo un óvalo.

	1	2	3	4	
In disagree	⬭	⬭	⬭	⬭	Strongly agree

77. SF3.6. I have been able to cope with the schoolwork burden related to my child(ren) without feeling overwhelmed.

Marca solo un óvalo.

	1	2	3	4	
In disagree	◯	◯	◯	◯	Strongly agree

Behavioral changes in children (from 6 months of age) during voluntary isolation.

If there are NO children living in your home, please go to the next section and complete the survey.

Please indicate if you have noticed any change in the behavior of children in your home since the outbreak began, by considering:

1. Disagree
2. Little agreement
3. Agree
4. Strongly agree

During the weeks we have been in the outbreak, in my house my child (ren) ...
Please complete according to your perception.

78. SF4.1. has(have) regular times for physical activities (sports, active games, dancing, etc.).

Marca solo un óvalo.

	1	2	3	4	
In disagree	◯	◯	◯	◯	Strongly agree

79. SF4.2. spends(spend) 30 minutes a day performing some type of physical activity (sports, active games, dancing, etc.).

Marca solo un óvalo.

	1	2	3	4	
In disagree	◯	◯	◯	◯	Strongly agree

80. SF4.3. has(have) regular times to go to sleep.

Marca solo un óvalo.

	1	2	3	4	
In disagree	◯	◯	◯	◯	Strongly agree

81. SF4.4 has(have) difficulty waking up in the morning.

Marca solo un óvalo.

	1	2	3	4	
In disagree	◯	◯	◯	◯	Strongly agree

82. SF4.5. has(have) difficulty sleeping at night.

Marca solo un óvalo.

	1	2	3	4	
In disagree	◯	◯	◯	◯	Strongly agree

83. SF4.6. has(have) regular schedules to make three meals each day.

Marca solo un óvalo.

	1	2	3	4	
In disagree	◯	◯	◯	◯	Strongly agree

84. SF4.7. has(have) changed the amount of food he or she (they) normally eat (eat more or eat less).

Marca solo un óvalo.

	1	2	3	4	
In disagree	◯	◯	◯	◯	Strongly agree

85. SF4.8. has(have) increased the time that he or she (they) spend in front of a screen (smartphone, tv, computer, tablet, etc.).

Marca solo un óvalo.

	1	2	3	4	
In disagree	◯	◯	◯	◯	Strongly agree

86. SF4.9. has(have) presented episodes of crying or anger more frequently and/or more easily than in normal conditions.

Marca solo un óvalo.

	1	2	3	4	
In disagree	◯	◯	◯	◯	Strongly agree

87. SF4.10. have (n) a certain time to do their homework.

Marca solo un óvalo.

	1	2	3	4	
In disagree	◯	◯	◯	◯	Strongly agree

88. SF4.11. has (n) a certain space to carry out their school tasks.

Marca solo un óvalo.

	1	2	3	4	
In disagree	◯	◯	◯	◯	Strongly agree

89. SF4.12. has(have) to do his or her (their) homework before playing freely.

Marca solo un óvalo.

	1	2	3	4	
In disagree	◯	◯	◯	◯	Strongly agree

90. SF4.13. School assignments left to my child (ren) during isolation have generated stress and anxiety responses in him (or them).

Marca solo un óvalo.

	1	2	3	4	
In disagree	◯	◯	◯	◯	Strongly agree

91. SF4.14. shows(show) difficulties related to starting an activity.

Marca solo un óvalo.

	1	2	3	4	
in disagree	⬭	⬭	⬭	⬭	Strongly agree

92. SF4.15. shows(show) difficulties related to completing an activity.

Marca solo un óvalo.

	1	2	3	4	
In disagree	⬭	⬭	⬭	⬭	Strongly agree

93. SF4.16. has(have) difficulties focusing on an activity (constantly distracted, getting up from the place where her or she (they) are carrying out an activity, etc.).

Marca solo un óvalo.

	1	2	3	4	
In disagree	⬭	⬭	⬭	⬭	Strongly agree

94. SF4.17. What are the most common types of play your child (ren) have during the period of voluntary isolation? (you can choose more than one).

Selecciona todos los que correspondan.

- [ ] My son is a baby so he prefers to play exploring (take objects, put them in his mouth, throw them, crawl, etc.)
- [ ] Running, jumping, sneaking, chasing, etc.
- [ ] Board games.
- [ ] Play at being a character (eg be a police officer, teacher, doctor, superhero, etc.).
- [ ] Videogames.
- [ ] Games with toys (cars, action figures, barbies, etc).
- [ ] Creative games (painting, modeling with clay, drawing, etc.).
- [ ] Prefer other recreational activities instead of playing (watching movies, videos, listening to music, surfing the internet etc.).

Otro: [ ] ____________________

95. SF4.18. What are the most frequent themes of play that you have observed in your child (ren) during the period of voluntary isolation? (you can choose more than one).

Selecciona todos los que correspondan.

- [ ] Violent games
- [ ] Fantasy games
- [ ] Games that represent current situations.
- [ ] Games that represent solidarity and help.

Otro: [ ] ____________________

96. SF4.19. During the period of voluntary isolation my child (ren) prefer to play or do recreational activities:

Selecciona todos los que correspondan.

- [ ] Alone
- [ ] With other children
- [ ] With adults

97. SF4.20. During the period of voluntary isolation I have noticed that my child (ren).

Selecciona todos los que correspondan.

- [ ] They play less time.
- [ ] They play the same time.
- [ ] They play more time.

Thank you very much for your help!

We remind you that within 30 days you will receive a second version of this questionnaire as a follow-up to this study.

98. If you agree to continue collaborating with our research, please leave an email.

____

Este contenido no ha sido creado ni aprobado por Google.

Google Formularios
